# Supplementary material for: 4-Mercaptopyridine-Modified Sensor for the Sensitive Electrochemical Detection of Mercury Ions
Source: Micromachines (Basel). 2023 Mar 27;14(4):739. doi: 10.3390/mi14040739 (PMC10143982; doi:10.3390/mi14040739)
Supplement: Supplementary file 1 [file micromachines-14-00739-s001.zip › micromachines-2264269-supplementary.pdf]

Supporting Information

4-Mercaptopyridine-Modified Sensor for the Sensitive Electrochemical Detection of Mercury Ions

Mingjie Han [1,2], Yong Xie [1,2], Ri Wang [1,2], Yang Li [1], Chao Bian [1,*] and Shanhong Xia [1,*]

[1] State Key Laboratory of Transducer Technology, Aerospace Information Research Institute,
Chinese Academy of Sciences, Beijing 100190, China; mjhan1993@outlook.com (M.H.); xieyong16@mails.ucas.ac.cn (Y.X.); wangri17@mails.ucas.ac.cn (R.W.); yangli@mail.ie.ac.cn (Y.L.)

[2] School of Electronic, Electrical and Communication Engineering, University of Chinese Academy of Sciences, Beijing 100190, China

* Correspondence: cbian@mail.ie.ac.cn (C.B.); shxia@mail.ie.ac.cn (S.X.)

Table of Contents

Figure S1. The electrostatic potential diagram of 4-MPY.
Figure S2. Repeatability and reproducibility study of the proposed sensor.
Figure S3. The change of the current intensity in the reusability experiment of 4-MPY/Au electrode with EDTA.
Table S1. The impedance fitting parameters of Au electrode surface at different modification conditions.

Table S2. Binding energy values of different interferents after reaction with 4-MPY.

Table S3. Stability constants of M^{2+}-pyridine complexes.

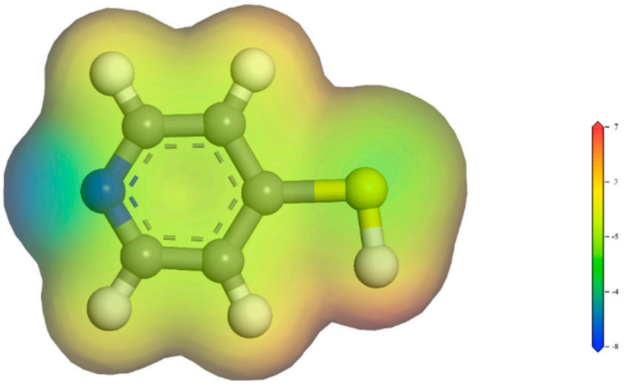

Figure S1. The electrostatic potential diagram of 4-MPY.

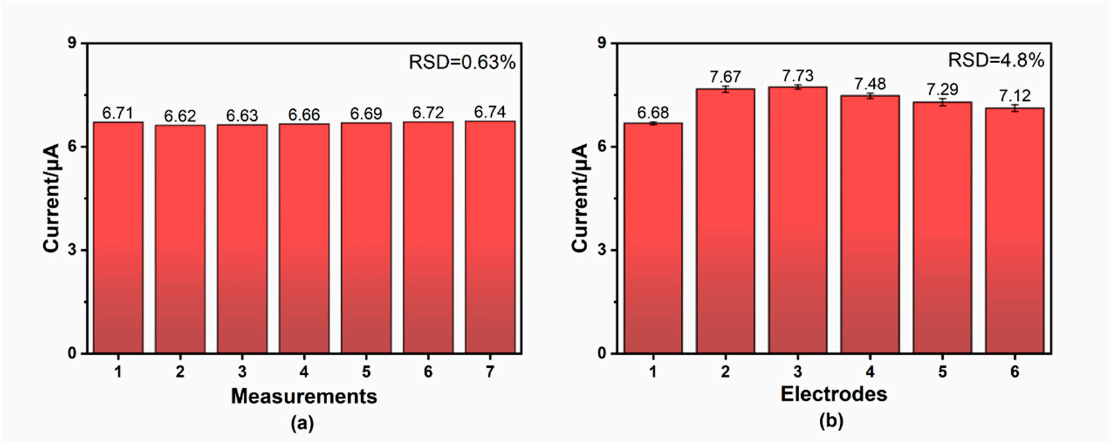

Figure S2. Repeatability and reproducibility study of the proposed sensor. (a) Current intensity in the repeatability experiment of 4-MPY/Au electrode for seven measurements (in 5 mM $[Fe(CN)_6]^{3-/4-}$ with 10 μg/L Hg^{2+}); (b) current intensity in the reproducibility experiment (in 5 mM $[Fe(CN)_6]^{3-/4-}$ with 10 μg/L Hg^{2+}).

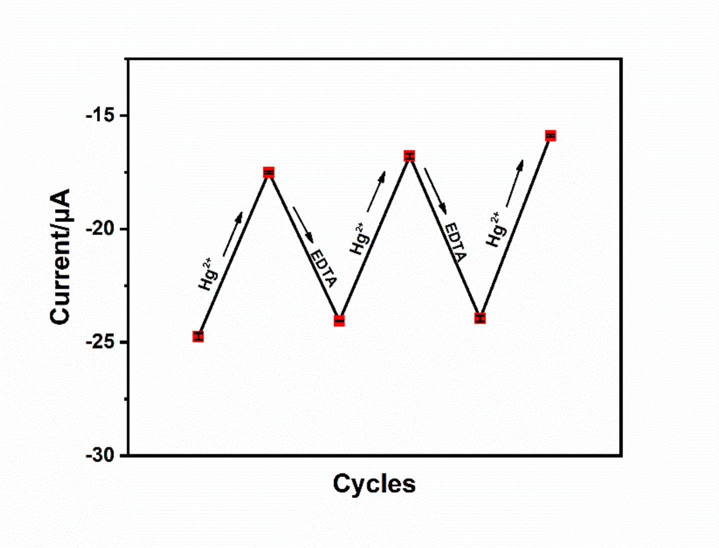

Figure S3. The change of the current intensity in the reusability experiment of 4-MPY/Au electrode with EDTA after reaction with 10 μg/L Hg2+.

Table S1. The impedance fitting parameters of Au electrode surface at different modification conditions.

	**R_s /Ω**	**R_{ct} /Ω**
Bare Au	212.5	125.8
4-MPY/Au	204.3	685.7
4-MPY/Au+Hg^{2+}	213.6	812.6

Table S2. Binding energy values of different interferents after reaction with 4-MPY.

Interferents	Complex	E_{M-Py} (Ha)	E_M (Ha)	E_{Py} (Ha)	BE (Ha[1])	BE (kcal/mol)
Hg^{2+}	MPY-Hg^{2+}-MPY	-1942.08	-1714.41	-227.139	-0.53002	-332.591
K^{+}	MPY-K^{+}	-1457.02	-857.211	-599.767	-0.04054	-25.441
Mg^{2+}	MPY-Mg^{2+}-MPY	-1914.07	-1714.41	-199.245	-0.4129	-259.101
Ca^{2+}	MPY-Ca^{2+}-MPY	-2391.61	-1714.41	-676.92	-0.28015	-175.798
Cu^{2+}	MPY-Cu^{2+}-MPY	-1936.5	-1714.41	-221.435	-0.64712	-406.074
Pb^{2+}	MPY-Pb^{2+}-MPY	-1834.57	-1714.41	-119.832	-0.32241	-202.314
Zn^{2+}	MPY-Zn^{2+}-MPY	-1968.4	-1714.41	-253.44	-0.55019	-345.25
Sn^{2+}	MPY-Sn^{2+}-MPY	-1836.45	-1714.41	-121.687	-0.35053	-219.96

[1] 1 Ha = 627.51kcal/mol

Table S3. Stability constants of M^{2+}-pyridine complexes.

M^{2+}	$\log K_1$	$\log K_2$	Ref
Hg^{2+}	5.1	4.9	1
Zn^{2+}	1.06	0.79	2
Cu^{2+}	2.56	1.89	3

[1] Bjerrum, J. METAL AMMINE FORMATION IN SOLUTION .15. SILVER(I)PYRIDINE AND MERCURY(II)PYRIDINE AND SOME OTHER MERCURY(II)AMINE SYSTEMS. *Acta Chem. Scand.* **1972**, *26*, 2734-&, doi:10.3891/acta.chem.scand.26-2734.

[2] Kurihara, M.; Ozutsumi, K.; Kawashima, T. COMPLEXATION OF MANGANESE(II) AND ZINC(II) IONS WITH PYRIDINE, 3-METHYLPYRIDINE AND 4-METHYLPYRIDINE IN DIMETHYLFORMAMIDE. *J. Chem. Soc.-Dalton Trans.* **1993**, 22, 3379-3382, doi:10.1039/dt9930003379.

[3]Tanaka, M.; Tabata, M. Stability Constants of Metal(II) Complexes with Amines and Aminocarboxylates with Special Reference to Chelation. *Bull. Chem. Soc. Jpn.* **2009**, *82*, 1258-1265, doi:10.1246/bcsj.82.1258.
